# Genetic Markers of Differential Vulnerability to Sleep Loss in Adults

**DOI:** 10.3390/genes12091317

**Published:** 2021-08-26

**Authors:** Courtney E. Casale, Namni Goel

**Affiliations:** Biological Rhythms Research Laboratory, Department of Psychiatry and Behavioral Sciences, Rush University Medical Center, 1645 W. Jackson Blvd., Suite 425, Chicago, IL 60612, USA; courtney_casale@rush.edu

**Keywords:** total sleep deprivation, sleep restriction, candidate genes, polymorphisms, circadian clock genes, interindividual differences, vulnerability, resilience, neurobehavioral, countermeasures

## Abstract

In this review, we discuss reports of genotype-dependent interindividual differences in phenotypic neurobehavioral responses to total sleep deprivation or sleep restriction. We highlight the importance of using the candidate gene approach to further elucidate differential resilience and vulnerability to sleep deprivation in humans, although we acknowledge that other omics techniques and genome-wide association studies can also offer insights into biomarkers of such vulnerability. Specifically, we discuss polymorphisms in adenosinergic genes (*ADA* and *ADORA2A*), core circadian clock genes (*BHLHE41/DEC2* and *PER3*), genes related to cognitive development and functioning (*BDNF* and *COMT*), dopaminergic genes (*DRD2* and *DAT*), and immune and clearance genes (*AQP4*, DQB1*0602, and *TNFα*) as potential genetic indicators of differential vulnerability to deficits induced by sleep loss. Additionally, we review the efficacy of several countermeasures for the neurobehavioral impairments induced by sleep loss, including banking sleep, recovery sleep, caffeine, and naps. The discovery of reliable, novel genetic markers of differential vulnerability to sleep loss has critical implications for future research involving predictors, countermeasures, and treatments in the field of sleep and circadian science.

## 1. Introduction

Located in the suprachiasmatic nuclei of the anterior hypothalamus, the biological clock, among other physiological processes, regulates the timing of sleep and wakefulness as well as waking behavior, creating circadian rhythmicity in neurobehavioral variables such as cognitive performance and sleepiness [1,2]. The two-process model of sleep regulation posits that a homeostatic process (Process S) and a circadian process (Process C) interact to modulate the timing of sleep onset and offset, as well as the stability of waking neurobehavioral functions [3,4,5,6,7]. Process S (the drive for sleep) increases while awake and decreases while asleep. Sleep onset occurs when the homeostatic drive increases above a certain threshold, and wakefulness is induced when it decreases below a different threshold [1]. Process C (the cycle of sleep and wakefulness) represents the daily oscillatory modulation of these thresholds and promotes wakefulness at certain times [8]. 

It is well established that sleep loss induces decrements in neurobehavioral functioning [4,9,10], and that there are robust, trait-like interindividual phenotypic differences related to the magnitude of such decrements, whereby some individuals are minimally affected by insufficient sleep (i.e., resilient) and others are greatly affected (i.e., vulnerable) [11,12,13,14,15,16,17,18].

**Figure 1:**
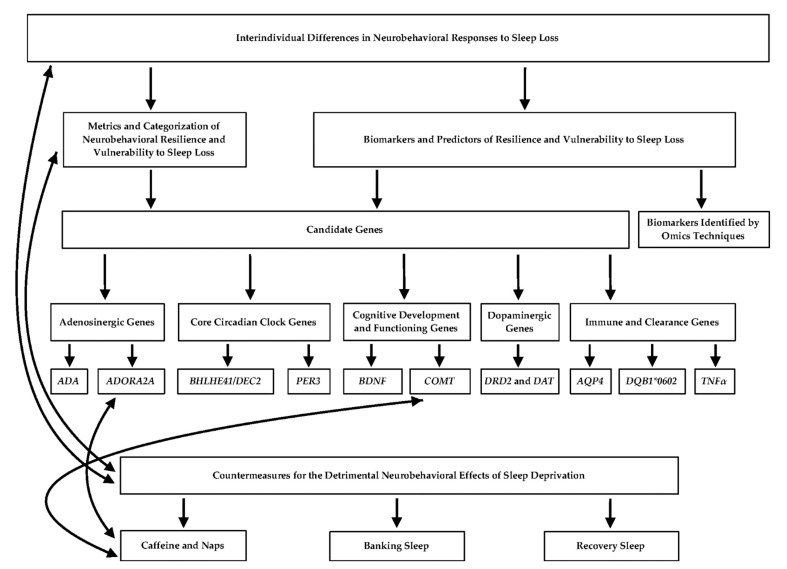
Flowchart depicting the relationships between the main concepts presented in the review. Solid black arrows indicate an established connection between the topics. Connections between genes and countermeasures are associative and not causal, thus warranting the need for further investigation.

This review explores the genetic underpinnings of phenotypic individual differences related to sleep deprivation, particularly in relation to differential neurobehavioral resilience and vulnerability. It also discusses the efficacy of various mitigation strategies for sleep loss-induced deficits including caffeine, naps, and recovery sleep, and examines candidate gene studies utilizing caffeine as a countermeasure. The review culminates with a discussion of future directions. Please refer to Figure 1 for a flowchart highlighting the main concepts discussed in this article and their relationships.

## 2. Interindividual Differences in Neurobehavioral Responses to Sleep Loss

### 2.1. Metrics and Categorization of Neurobehavioral Resilience and Vulnerability to Sleep Loss

Common metrics of neurobehavioral functioning for classifying vulnerable and resilient individuals include behavioral attention tasks such as the Psychomotor Vigilance Test (PVT) [19], cognitive throughput tasks such as the Digit Symbol Substitution Test [20], working memory tests such as n-back tasks [21] and the Digit Span Task [22], measures of self-rated sleepiness such as the Karolinska Sleepiness Scale (KSS) [23], and measures of self-rated fatigue, vigor, and mood such as those derived from the Profile of Mood States (POMS) [24]. Importantly, numerous studies have reported that an individual’s resilience or vulnerability to sleep loss when assessing performance on objective and self-rated metrics are not related [12,17,18,25,26], making the determination of reliable categorization methods more complex. The use of a variety of neurobehavioral tests when conducting individual differences research mitigates this issue and allows for better cognitive endophenotyping (accurately capturing the complete essence of a cognitive phenotype through targeted measurements) [27].

Several approaches have been used to classify individuals as resilient or vulnerable in past research, although the optimal methods to do so remain unknown. The most common prior approaches either utilized raw performance or self-rated scores on neurobehavioral tasks during sleep deprivation [15,28,29,30,31] or utilized difference scores that accounted for baseline performance [32,33,34,35,36,37,38]. Intraindividual variance, which considers time-of-day variation in performance [9,39,40,41,42,43,44], has been posited as another potential method to characterize resilience or vulnerability to sleep deprivation. However, further research is needed regarding this approach, since only one published study using two commonly used cognitive measures has explicitly investigated intraindividual variation as a categorization method [45]. Furthermore, the threshold used to divide individuals into resilient and vulnerable groups has also varied in prior research—studies have utilized a median split [28,29,30,32,35,36,46,47,48,49,50], a tertile split [15,34,38,51], a quartile split [31,52], or other numeric divisions of neurobehavioral performance [33,53]. Nevertheless, although more research is needed to determine consistent categorizations and predictors of resilience and vulnerability based on neurobehavioral performance, it remains important to explore possible biological indicators of such characteristics.

### 2.2. Biomarkers and Predictors of Resilience and Vulnerability to Sleep Loss

While definitive predictors of neurobehavioral resilience and vulnerability to sleep loss have yet to be discovered, genetic and omics (e.g., epigenomic, transcriptomic, metabolomic, and proteomic) techniques have identified biomarkers (objective proxies of biological processes that allow for remote detection of such processes, regardless of their mechanistic role in the assessed condition [54]) to differentiate an individual’s response to chronic sleep restriction (SR; several consecutive nights with a reduction in total sleep time) or total sleep deprivation (TSD; one or more nights without sleep) [4]. While numerous biomarkers and other factors, such as neurobehavioral performance, are considered potential predictors of differential responses to sleep loss, genetic polymorphisms (variants in DNA sequence) are perhaps one of the most studied indicators (see Figure 1).

## 3. Genetic Polymorphisms Related to Differential Neurobehavioral Vulnerability to Sleep Loss

Neurobehavioral vulnerability to sleep loss is a heritable and stable trait. One twin study found substantial differential neurobehavioral vulnerability to acute TSD (as measured by PVT performance), with 56.2% of the total variance in monozygotic twins, and only 14.5% of the variance in dizygotic twins, attributable to variance between pairs of twins [55]. This study [55] and other studies using unrelated participants [56] support the notion that an individual’s neurobehavioral response to sleep deprivation is a genetically determined and phenotypic trait. Additionally, although neurobehavioral performance during sleep loss is typically normally distributed [13,57] and suggests a polygenetic phenotype [4], previous candidate gene studies have found several specific genetic polymorphisms that are associated with differential neurobehavioral responses to sleep loss. The candidate gene approach is useful for determining the influence of genetic variants on neurobehavioral performance during sleep-deprived and rested conditions. Genome-wide association (GWA) studies have also revealed findings in support of trait-like interindividual differences in sleep parameters and may be useful in determining neurobehavioral vulnerability in response to sleep deprivation (reviewed in [58]). Furthermore, techniques that incorporate a perturbation of the system (e.g., enforced sleep deprivation), theory-driven genotyping, selective sampling, and cognitive endophenotyping are particularly valuable for studying differential vulnerability in relatively small samples [27]. Below, we summarize the genetic underpinnings of differential neurobehavioral responses to sleep loss that explain a portion of the related interindividual variance in resilience and vulnerability. We refer the reader to Goel [58], Yamazaki and Goel [59], Dutta [60], Bolsius et al. [61], and Garfield [62] for reviews of candidate gene and GWA studies related to sleep parameters during rested conditions, normal sleep, and circadian rhythm sleep-wake disorders, and we refer the reader to Goel [54], Mullington et al. [63], and Uyhelji et al. [64] for reports describing additional biomarker and omics techniques used in the context of sleep loss, which are beyond the scope of this article.

### 3.1. Adenosinergic Genes

#### 3.1.1. *ADA*

The enzyme adenosine deaminase (ADA) breaks down and regulates intra- and extracellular adenosine levels. Studies have demonstrated differential neurobehavioral vulnerability to sleep deprivation associated with the *ADA* G22A polymorphism (single nucleotide polymorphism (SNP) ID: rs73598374). Bachmann et al. [65] found that *G/A* participants had worse vigilant attention, as measured by PVT lapses (>500 ms reaction time) and PVT response speed, and greater self-reported sleepiness and fatigue compared to participants with the *G/G* genotype [65]. However, in a large twin sample, this *ADA* polymorphism did not relate to differential vulnerability to TSD, as defined by PVT performance [55]. Another study [66] used a protocol consisting of two sleep conditions (either 40 h TSD or 40 h multiple naps) to investigate the influence of this *ADA* polymorphism on neurobehavioral measures under conditions of high versus low sleep pressure. The authors found that *G/A* individuals reported greater subjective sleepiness than *G/G* individuals only during TSD when sleep pressure was high. *G/A* individuals also reported worse subjective wellbeing during TSD than during the nap condition and exhibited worse working memory performance when sleep pressure was high [66]. Furthermore, at the end of the night, *G/G* individuals performed better on the PVT during the nap condition, when sleep pressure was low, than during TSD [66].

#### 3.1.2. *ADORA2A*

The T1083C polymorphism (SNP-ID: rs5751876) in the adenosine A2A receptor (*ADORA2A*) gene also contributes to an individual’s neurobehavioral vulnerability to sleep loss: a recent study found that *T* allele carriers had worse vigilant attention performance (greater number of PVT lapses) than *C/C* individuals after 32 h TSD [67]. Similarly, another study showed that *C/T* carriers exhibited greater PVT performance resilience during chronic SR compared with *T/T* carriers [68]. Additionally, out of the eight distinct haplotypes created by eight SNPs in *ADORA2A* (SNP-IDs: rs5751862, rs5760405, rs2298383, rs3761422, rs2236624, rs5751876, rs35320474, and rs4822492), HT4 allele carriers performed better on the PVT after a night of TSD than those without the HT4 haplotype [69]. 

Moreover, the *ADORA2A* polymorphism is particularly integral to an individual’s sensitivity to caffeine, and accordingly plays a role in whether caffeine may be an especially effective mitigation strategy for maintaining neurobehavioral functioning during extended wakefulness. Caffeine is structurally similar to adenosine, thus contributing to its stimulating properties during sleep loss [70]. One study found that caffeinated coffee mitigated performance deficits on tasks of vigilant attention and executive control, but not self-rated sleepiness, induced by five consecutive nights of SR in *C/C* allele carriers [71]. Furthermore, caffeine counteracted 40 h TSD-induced decrements in PVT performance in non-HT4 allele carriers but was not effective in individuals with the HT4 haplotype [69], although another study showed that the *ADORA2A* genotype did not influence the mitigatory effect of caffeine on PVT performance during 38 h TSD [67]. In addition, although A_1_ adenosine receptor (A_1_AR) availability has been linked to the mitigatory effects of recovery sleep on neurobehavioral performance following 52 h TSD, this effect was not moderated by the *ADORA2A* genotype [72]. These studies have particularly important implications for determining whether mitigation strategies involving caffeine consumption may be more beneficial to certain individuals based on their *ADORA2A* genotype. Please see the Section 4 entitled, “Countermeasures for the Detrimental Neurobehavioral Effects of Sleep Deprivation” for further discussion of this concept (also see Figure 1).

### 3.2. Core Circadian Clock Genes

#### 3.2.1. *BHLHE41/DEC2*

The *BHLHE41/DEC2* gene is involved in the circadian regulation of sleep quantity, and also contributes to differential vulnerability to sleep loss. Variants of *BHLHE41/DEC2* have been reported to be involved in responses to chronic SR and acute TSD [73]. In this study with one pair of twins, the presence of the Y362H variant (c.1084C>T; no SNP-ID available) was associated with fewer lapses on the PVT during TSD, reduced sleep duration, and less recovery sleep following TSD, though non-rapid eye movement sleep duration did not differ between the twins [73]. The Y362H variant also decreased the ability of *BHLHE41/DEC2* to suppress in vitro CLOCK/BMAL1 and NPAS2/BMAL1 transactivation [73]. The differential neurobehavioral resilience to sleep deprivation related to *BHLHE41/DEC2* may be a consequence of a decreased need for sleep, as the P384R mutation (SNP-ID: rs121912617) has been implicated in familial natural short sleep [74].

#### 3.2.2. *PER3*

*PER3*, a core circadian clock gene, has a 54-nucleotide coding region variable-number tandem repeat (VNTR) (SNP-ID: rs57875989) repeating in either four or five units that is associated with differential vulnerability to sleep deprivation. TSD studies have shown that individuals with the *PER3^5/5^* genotype (five-repeat allele) had worse cognitive performance [75], poorer executive function performance selectively in the early morning [76], and greater decrements in sustained attention [77] compared to *PER3^4/4^* (four-repeat allele) individuals. However, the *PER3* genotype did not differentially influence neurobehavioral vulnerability to acute TSD in twins [55] or to chronic SR in unrelated individuals [78]. In contrast to Goel et al. [78], two other studies found individual differences in neurobehavioral responses to SR related to this *PER3* VNTR polymorphism [68,79].

The *PER3* C7827519G SNP (SNP-ID: rs228697) has also been studied in relation to interindividual differences in responses to sleep loss. However, a study involving 38 h TSD found neither an association between *PER3* genotype and neurobehavioral response to TSD, nor an interaction between *PER3* genotype, TSD, and caffeine on PVT performance at any point throughout the protocol [67].

### 3.3. Cognitive Development and Functioning Genes

#### 3.3.1. *BDNF*

The brain-derived neurotrophic factor (*BDNF*) gene is essential for proper neuronal development and neuronal plasticity, including as applied to neurobehavioral functioning. The *BDNF* Val66Met polymorphism (SNP-ID: rs6265) has been found to impact an individual’s vulnerability to sleep deprivation, as studies have shown that *Met* carriers performed more poorly on neurobehavioral metrics than *Val/Val* individuals during extended wakefulness. One study demonstrated that *Met* carriers had worse response inhibition performance on the Stroop Task, which assesses cognitive flexibility, than *Val/Val* homozygotes after 20 h of wakefulness, suggesting differential impairment related to this *BDNF* polymorphism [80]. Similarly, *Met* carriers also performed more poorly than *Val/Val* homozygotes on a verbal two-back working memory task after 40 h of wakefulness [81]. However, this *BDNF* genotype did not differentially impact sustained attention, or self-rated sleepiness or wellbeing [81]. In contrast, a study involving simulated night shift schedules demonstrated that the *Val/Met BDNF* genotype may allow for greater flexibility to adapt to circadian misalignment, since heterozygotes showed fewer lapses on the PVT toward the end of the night shifts in the second simulation bout as compared to the first bout; *Val/Val* homozygotes did not show a performance difference between night shift bouts [82]. Taken together, these findings suggest possible genotypically regulated differences in BDNF protein expression during sleep loss, which also may be important for determining neurobehavioral resilience and vulnerability [83].

#### 3.3.2. *COMT*

The catechol-O-methyltransferase gene (*COMT*) encodes the COMT protein, which is responsible for breaking down catecholamines including epinephrine, norepinephrine, and dopamine. The functional *COMT* Val158Met polymorphism (SNP-ID: rs4680) has been found to be associated with neurobehavioral responses to sleep deprivation. After a night of TSD, individuals with the *Val* allele demonstrated poorer performance on tasks of behavioral attention [84] and adaptive decision making [85] than *Met* carriers. During chronic SR, however, this *COMT* polymorphism did not differentially impact cognitive performance or subjective or physiological sleepiness [86].

Additionally, this *COMT* polymorphism was shown to regulate the effect of modafinil, a pharmacological stimulant, on behavioral attention, self-reported wellbeing, and executive functioning, yet was not associated with subjective sleepiness [87,88]. A recent study also investigated the potential effect of this *COMT* polymorphism on performance impairment during TSD both independent from, and related to, the mitigatory effect of caffeine. While no main effect of *COMT* genotype was observed on PVT performance or KSS scores overall, the study found that, after 20 h TSD, but not after 26 h or 32 h TSD, *Met* allele carriers had more PVT lapses than *Val/Val* individuals in the placebo condition [67]. Notably, this genotypic performance difference was not observed when participants were provided caffeine, suggesting its beneficial impact [67]. Please see the Section 4 entitled, “Countermeasures for the Detrimental Neurobehavioral Effects of Sleep Deprivation” for more details about the potential link between genetic polymorphisms and mitigation strategies (also see Figure 1).

### 3.4. Dopaminergic Genes

#### *DRD2* and *DAT*

The dopamine D2 receptor gene (*DRD2*) C957T polymorphism (SNP-ID: rs6277) and the dopamine transporter gene (*DAT1*) 3′-UTR VNTR polymorphism (SNP-ID: rs28363170) have also been implicated in differential cognitive vulnerability to sleep deprivation, both separately and in combination with each other. During 38 h TSD, *DRD2 C/C* individuals were particularly resilient to the effects of sleep loss on cognitive flexibility, whereas *T/T* individuals were particularly vulnerable; however, no genotypic influence was found on PVT performance resilience [89]. Another TSD study showed that *DRD2 T/T* homozygotes demonstrated greater declines in performance with increased time spent performing the PVT than *C* allele carriers, suggesting that the *DRD2* genotype predicts the magnitude of this time-on-task (TOT) effect [90]. Similarly, the *DAT1* genotype was also found to modulate the TOT effect on the PVT during TSD, as 10-repeat allele (*10R*) homozygotes showed less severe TOT deficits compared to nine-repeat allele (*9R*) carriers [91]. Additionally, when examining the combination of these two polymorphisms, individuals with the *DAT1*-*DRD2 10R*/*10R*-*C/T* or *9R*-*C/C* genotypes showed greater PVT performance resilience from TSD-induced decrements than individuals with other genotype combinations [92]. *DAT1*-*DRD2 9R*-*C/C* individuals were also most resistant to self-reported sleepiness [92]. Collectively, these studies exemplify the influence of multiple different genetic polymorphisms on individual responses to sleep deprivation, as well as suggest a modulatory effect of dopaminergic pathways on some neurobehavioral responses to sleep loss.

### 3.5. Immune and Clearance Genes

#### 3.5.1. *AQP4*

Aquaporin 4 (AQP4) is an astrocytic water channel that facilitates the flow of cerebrospinal fluid (CSF) throughout the brain. The *AQP4* gene has several SNPs that modulate the expression of AQP4, and which were recently associated with differential responses to 40 h TSD (SNP-IDs: rs162007, rs162008, rs63514, rs455671, rs335931, rs335930, rs335929, and rs16942851). Ulv Larsen et al. [93] reported that individuals with the low-AQP4-expressing HtMi variant haplotype (carriers of the minor allele) had less of a reduction in PVT response speed and less of an increase in self-rated sleepiness than individuals with the HtMa haplotype (carriers of the major allele) during prolonged wakefulness, suggesting that HtMi individuals may be more neurobehaviorally resilient to sleep deprivation [93]. These findings are particularly important for the relationship between CSF flow and brain clearance since these are essential for protecting against the development of neurodegenerative diseases.

#### 3.5.2. DQB1*0602

The human leukocyte antigen DQB1*0602 allele, which relates to narcolepsy [94,95], has been investigated in relation to differential vulnerability to sleep loss. During five consecutive SR nights, DQB1*0602-positive individuals reported greater subjective sleepiness than DQB1*0602-negative individuals [96]. Similarly, DQB1*0602-positive individuals reported greater self-rated sleepiness and fatigue during baseline [96]. Additionally, during SR, DQB1*0602-positive individuals exhibited differentially greater increases in subjective fatigue [96]. Notably, carrying the DQB1*0602 allele did not differentially influence cumulative decrements in neurobehavioral performance induced by SR, as total decreases in cognitive performance and increases in physiological sleepiness were comparable between both positive and negative groups [96].

#### 3.5.3. *TNFα*

The tumor necrosis factor alpha gene (*TNFα*) encodes TNFα, a proinflammatory cytokine, and has a G308A polymorphism (SNP-ID: rs1800629) in its promoter region. This polymorphism has been associated with differential resilience to sleep-loss-induced deficits on the PVT, as *A* allele carriers demonstrated greater performance resilience during TSD [97,98]. Additionally, one study demonstrated a greater sensitivity of *TNFα A* allele carriers to the effects of caffeine than *G/G* carriers during TSD; however, this effect may be related to greater TSD performance degradation in *A* allele carriers since PVT lapses did not differ as a function of genotype [67]. However, Skeiky et al. [98] did not find an interaction of *TNFα* genotype and caffeine (either 200 or 300 mg doses) on PVT performance during 48 h TSD, despite demonstrating a genetic influence on PVT performance variance alone.

### 3.6. Strengths and Weaknesses of the Candidate Gene Approach

The candidate gene approach for studying differential vulnerability to sleep deprivation, largely driven by the existence of phenotypic individual differences in neurobehavioral responses to sleep loss, has been commonly used to investigate the influence of genetic variants on such trait-like responses [4]. This approach is useful for investigating the association between specific genetic polymorphisms and phenotypic responses to sleep loss, particularly in a laboratory setting with relatively small sample sizes. Although the candidate gene approach is useful, no published studies have used this approach to explicitly determine causality rather than associations between genotype and phenotype. Nevertheless, previous work has demonstrated that the influence of some of the aforementioned SNPs, individually or in combination, may explain a substantial portion of the variance in neurobehavioral responses to sleep loss (reviewed in Satterfield et al. [27]). Thus, it is important to continue using the candidate gene approach to examine the contribution of specific genes to differential neurobehavioral vulnerability.

## 4. Countermeasures for the Detrimental Neurobehavioral Effects of Sleep Deprivation

The neurobehavioral and physiological effects of sleep loss are detrimental yet often are undetected by sleep-deprived individuals. Although the optimal way to protect against poorer neurobehavioral performance and adverse health outcomes that are associated with sleep deprivation is to consistently obtain sufficient sleep aligned with an individual’s circadian rhythms [99], societal and work demands often make this difficult to achieve. This is especially true for populations such as shift workers, students pulling “all-nighters”, on-call medical personnel, transmeridian travelers, and individuals whose jobs require extended wakefulness [9]. Sleep deprivation also directly impacts driving and accident risk—sleepiness-related crashes exhibit similar injury and fatality rates as alcohol-related crashes [100,101,102], though they are often underestimated [9,103,104,105,106]. Thus, mitigation strategies to combat the severity of such negative effects, including banking sleep, recovery sleep, caffeine, and naps, are critical. It is particularly important to investigate the efficacy of countermeasures in relation to the aforementioned candidate genes, as doing so may offer more definitive recommendations as to which individuals would benefit most from certain mitigation strategies based on their genetic make-up (see Figure 1).

### 4.1. Effects of Banking Sleep on Neurobehavioral Performance

Banking sleep—increasing sleep duration to 8–9 h per night for several consecutive nights—has been demonstrated to mitigate neurobehavioral decrements resulting from subsequent sleep loss, including diminished performance on sustained attention tasks [107,108] and high-cognitive-load decision tasks [109]. Banking sleep also has been reported to effectively manage fatigue, stress, and excessive daytime sleepiness [110,111], which may be useful in applied settings such as military operations [112] and shift work [113]. The beneficial effects of banking sleep have been found to persist during a recovery sleep opportunity following sleep deprivation [107,108]. Though promising, further research is needed to establish whether this strategy can reliably maintain neurobehavioral performance during sleep-deprived conditions and subsequent recovery, especially in relation to interindividual phenotypic and genotypic differences. Further research is also necessary to determine whether the utility of banking sleep may be greater for certain individuals; for example, although not yet examined, the *BHLHE41/DEC2* polymorphism has been implicated in short sleep [74], which suggests that increasing sleep through banking may not be as effective for individuals with the P384R mutation.

### 4.2. Effects of Recovery Sleep on Neurobehavioral Performance

Recovery sleep—increased nightly sleep opportunity following a period of sleep deprivation—has also been proposed as a mitigation strategy to facilitate the restoration of several neurobehavioral measures after sleep loss. Some studies have shown that recovery sleep improved cognitive performance, reduced sleepiness, fatigue, and sleep propensity, increased alertness, and improved mood [114,115,116,117]. However, other studies have found that recovery sleep failed to completely reverse sleep deprivation-induced performance impairments on vigilance and working memory tasks, worsened inhibition as defined by a pinball task, and decreased self-rated vigor as defined by the POMS [10,117,118]. While reliable biomarkers of response to recovery sleep have yet to be discovered, interindividual differences may account for some of the discrepancies in research related to recovery sleep, since differential vulnerability could impact the amount of recovery sleep needed for certain aspects of neurobehavioral functioning to return to baseline levels [117]. Thus, it is important to further investigate biomarkers and genetic polymorphisms that may underlie differences in the effectiveness of recovery sleep.

### 4.3. Effects of Caffeine and Napping on Neurobehavioral Performance

The efficacy of caffeine in attenuating neurobehavioral performance deficits induced by sleep loss has been well established [119,120,121,122,123,124]. Acute caffeine consumption (using doses from <80 mg to 600 mg) has been shown to mitigate performance declines in sleep-deprived individuals in a variety of domains, including on attention, memory, information processing, executive functioning, and driving tasks (reviewed in [125]). As aforementioned, the efficacy of caffeine has also been linked to the *ADORA2A* [69,71] and *COMT* [67] genotypes, thus, further evincing caffeine’s biological utility. Notably, caffeine becomes less effective at preventing performance declines as the pressure for sleep increases during extended wakefulness [4]. In addition, robust individual differences in response to both sleep deprivation and caffeine confound the effectiveness of this mitigation strategy [4,123,124]. 

Napping during the day is another effective countermeasure to prevent performance declines in conditions of increased sleepiness and decreased alertness [126]. Although naps are beneficial, rest opportunities are typically followed by sleep inertia (a period of grogginess and diminished performance) upon awakening [127]. This has traditionally been thought to be especially true after longer naps during which slow-wave sleep is reached, though recent reports showed mixed findings [128]. Since naps alone are also unable to prevent the negative effects of sleep deprivation under all conditions [4,123,127] and across all neurobehavioral domains [129,130], the combination of caffeine consumption and a short nap may provide maximum protection against sleep-loss-induced decrements [127,131].

## 5. Conclusions and Perspectives

Adequate sleep is a biological imperative, essential for maintaining waking neurobehavioral performance, though it is often difficult to achieve. Thus, determining reliable predictors of differential vulnerability to sleep loss is crucial, given that diminished neurobehavioral functioning may negatively impact productivity and performance in a variety of real-world settings. As discussed, past research has identified several candidate genes and genetic polymorphisms related to circadian factors, neurotransmitter transmission, and immune and cognitive functioning, among others, which are associated with neurobehavioral resilience and vulnerability to sleep deprivation [67,80,82,84,85,89,90,91,93,98] (also see review [4]), and which are important components of individual differences research. Notably, some studies have also identified genetic polymorphisms involved in the efficacy of specific countermeasures used for sleep loss-induced deficits such as caffeine [67,69,71,98] (also see review [4]), suggesting that particular countermeasures may be more effective for certain individuals based on their own genetic profile. Importantly, establishing causality between specific genes and mitigation strategies through the candidate gene approach will enable the implementation of more individualized approaches for countering sleep loss-induced deficits, which is especially important for maximizing neurobehavioral functioning in applied settings.

It also is important to investigate the genetic determinants of resilience and vulnerability in diverse demographic and clinical populations. Studies have reported the influence of ethnicity and/or race on sleep characteristics [132,133,134,135], which are likely impacted by genetic ancestry and social and environmental pressures. Additionally, although associations between various candidate genes (e.g., *ADA*, *ADORA2A*, *PER3*) and clinical and/or sub-clinical symptomology and conditions have been shown [136,137,138,139,140], genotypic relationships between such symptomology and neurobehavioral performance have not been directly examined in the context of sleep loss. Interindividual differences in self-rated personality traits have also been proposed as factors contributing to differences in sleep characteristics [141,142] and to differential vulnerability to sleep loss [143]; however, the polygenetic and complex nature of personality makes it difficult to conduct genetic studies exploring this relationship. Further research on the genetic underpinnings of neurobehavioral responses to sleep deprivation is necessary to create a more generalizable framework of resilience and vulnerability to sleep loss-induced decrements. Overall, investigating such topics will lead to the development of personalized countermeasures and treatments based on an individual’s genetic and neurobehavioral performance profiles, which is critical for optimizing functioning in applied settings involving extended wakefulness.

## Data Availability

No new data were created in this study. Data sharing is not applicable to this article.
